# The relationship between the orientation of the lateral decubitus position for spinal anesthesia and positioning pain in patients with a femoral neck fracture: randomized non-inferiority trial

**DOI:** 10.1186/s40981-023-00595-y

**Published:** 2023-01-27

**Authors:** Keisuke Yoshida, Itaru Hareyama, Yoshie Noji, Shiori Tanaka, Kazuhiro Watanabe, Satoki Inoue

**Affiliations:** 1 Department of Anesthesiology, Aidu Chuo Hospital, 1-1, Tsuruga-machi, Aizuwakmatsu, Fukushima 965-8611 Japan; 2 Department of Anesthesiology, Fukushima Medical University, 1, Hikariga-oka, Fukushima, 960-1297 Japan

**Keywords:** Spinal anesthesia, Positioning pain, Lateral decubitus position, Hip fracture, Femoral neck fracture

## Abstract

**Background:**

To date, no clinical studies have investigated the relationship between positioning pain and orientation of the lateral decubitus position for hip fracture surgery. The aim of the present study was to test the hypothesis that performing spinal anesthesia in the lateral decubitus position with the fracture side up or down does not affect positioning pain in patients with a femoral neck fracture.

**Methods:**

This single-center, prospective, randomized non-inferiority trial examined 78 patients who received surgery for a femoral neck fracture under spinal anesthesia. By performing spinal anesthesia in the left lateral decubitus position in all patients, the positioning of the fracture up or down was randomized. Pain score during spinal anesthesia was evaluated objectively (0, calm; 1, facial grimacing; 2, moaning; 3, screaming; or 4, unable to proceed because of restlessness or agitation).

**Results:**

The data from 66 patients (fracture side down [*n* = 35] and up [*n* = 31]) were analyzed. There were no significant differences between the fracture side down and fracture side up groups regarding the percentage of patients who were assessed to have intense pain (score ≥ 3) when changing position from the supine to lateral position (13/35 [37%] vs 12/31 [39%]; 95% confidence interval [95% CI] for the difference of the percentage of patients of intense pain between the groups − 25.0 to 2.2; *p* = 1.000).

**Conclusions:**

There were no significant differences in the percentage of patients experiencing severe pain between the two groups. The 95% CI exceeded the preliminarily set a margin of inferiority of 20%; thus, the present study could not demonstrate the non-inferiority of the fractured side down group in terms of pain score.

## Background

With the aging of the global society, the number of operations for hip fractures (for example, femoral neck fractures and femoral trochanteric fractures) has increased [1]. Although no consensus has been reached on whether general anesthesia or regional anesthesia is optimal for hip fracture surgery [2–5], many institutions mainly perform spinal anesthesia, especially in the coronavirus pandemic [6].

When performing spinal anesthesia for hip fracture surgical repair, although few facilities perform anesthesia in the sitting position, many facilities perform it in the lateral decubitus position, especially with the fracture side facing up [7–11]. In order to perform spinal anesthesia, it is necessary to change the patient’s position from supine to lateral decubitus position, but many patients complain of severe pain when making such changes. Not only is this pain distressing for the patients, but it is also dangerous for compromise high-risk cardiac patients; thus, measures to reduce the pain during spinal anesthesia have been desired. To date, to reduce positioning pain, the effectiveness of a nerve block [7–10, 12, 13] and intravenously administered drugs such as fentanyl, ketamine, and dexmedetomidine [7–10, 14] have been investigated. Although these have some effect on reducing positioning pain, they take some effort and time, and there is a risk of side effects such as nerve injury, local anesthetic systemic toxicity, and respiratory depression, especially in elderly people. At our facility, considering that the patient population is quite elderly, we have not used these methods.

Regarding positioning pain for spinal anesthesia, we turned our attention to the orientation of the lateral decubitus position. At our facility, spinal anesthesia is usually performed with the patient in the lateral decubitus position with the fracture side up, which is consistent with many other facilities. However, although the conventional method of performing spinal anesthesia is with the patient in the lateral decubitus position with the fracture side up, to the best of our knowledge there is no scientific evidence to validate it. Of particular note, no clinical studies have investigated the relationship between positioning pain and orientation of the lateral decubitus position in hip fracture surgery. Thus, we hypothesized that performing spinal anesthesia in the lateral decubitus position with the fracture side up or down does not affect positioning pain. If non-inferiority of the lateral decubitus position with the fracture side down is shown, using hyperbaric local anesthetic and performing spinal anesthesia in the anesthesiologist’s preferred orientation are also effective options. The aim of this randomized non-inferiority trial was to test this hypothesis in patients with a femoral neck fracture.

## Methods

### Study design

This prospective randomized non-inferiority trial in a single-center was approved by the Ethics Committee of Aidu Chuo Hospital (approval number: 2013, Aizuwakamatsu, Japan), and the trial was registered at the University Hospital Medical Information Network Clinical Trials Registry (UMIN000043694, https://upload.umin.ac.jp/cgi-open-bin/ctr_e/ctr_view.cgi?recptno=R000049892, registered on March 24, 2021) prior to patient recruitment. The trial was conducted and reported according to the Consolidating Standards of Reporting Trials (CONSORT) 2010 statement [15].

The study was conducted at the Department of Anesthesiology in Aidu Chuo Hospital, Aizuwakamatsu, Japan. Seventy-eight patients who were scheduled for unilateral femoral neck fracture surgery, that is, femoral head prosthetic replacement, between March 2021 and August 2022 gave written informed consent to participate in the study. The inclusion criteria were (1) an age of ≥ 20 years; and (2) scheduled to undergoing surgery under spinal anesthesia. The exclusion criteria were (1) judgement by the orthopedic surgeon that the metastasis of the fracture was so severe that the position may lead to exacerbation of metastasis; (2) atrial fibrillation; (3) taking a beta blocker; (4) little-to-no pain in the fracture area (e.g., use of analgesics within 6 h of the beginning of surgery, sensory paralysis at the fracture site); (5) inability to get into the left lateral decubitus position for reasons other than pain; (6) trauma other than the unilateral hip fracture; and/or (7) judgement by the attending anesthesiologist that spinal anesthesia impossible.

### Randomization

To the best of our knowledge, there is no left-right difference in the incidence of femoral neck fractures. Thus, by performing spinal anesthesia in the left lateral position in all 66 patients, randomization of the patients regarding whether the fracture side was up or down was natural. Due to the design of this study, it could not be blind.

### Spinal anesthesia protocol

Anesthetic and surgical procedures were performed in the usual manner at our hospital. No patient was administered premedication. A peripheral or central venous line was secured in the ward before surgery. After the patient was brought into the operating room on a stretcher, standard monitors, such as a pulse oximeter, electrocardiograph, and non-invasive blood pressure, were applied in the supine position, and the measurements were recorded every 2.5 min.

All enrolled patients were placed in the left lateral decubitus position by 4–5 staff members (an anesthesiologist, an orthopedic surgeon, and 2 or 3 operating room nurses), paying as much attention to the fractured part as possible. When changing the position, the patient’s head was held by the anesthesiologist, and the fractured lower limb was held by the orthopedic surgeon. Then, an operating room nurse flexed the patient’s neck and held the patient in the left lateral decubitus position with the non-fractured side hip flexed.

After disinfection of the puncture site and draping, local anesthesia was applied to the puncture site with around 3 mL of 1% lidocaine using a 25-guage needle. All patients received spinal anesthesia with around 15 mg (3.0 mL) of 0.5% isobaric bupivacaine by either of the two experienced right-handed authors (I.H. or Y.N.). Spinal anesthesia was performed from the intervertebral space of L3/4 or L2/3 in the left lateral decubitus position by using a 25-guage needle (TOP Spinal Needle, TOP Corp., Tokyo, Japan). Three minutes after spinal injection, an anesthetic effect range was confirmed by evaluating the loss of cold sensation, after confirming that the range reached around T10 bilaterally. Then, the patient was placed in the surgical position and the surgery was started. The anesthetic effect range was also confirmed at the end of surgery.

### Outcome measures

Each patient’s pain score and heart rate were recorded by an operating room nurse at four time points: (1) when entering the room in the supine position (baseline); (2) when changing position to the left lateral decubitus position; (3) when local anesthesia was started; and (4) when puncturing spinal injection was successful. The pain score used was based on a scale reported by Lee et al. [14], that is, a scale of from 0 to 4 (0, calm; 1, facial grimacing; 2, moaning; 3, screaming; and 4, unable to proceed because of restlessness or agitation). In addition, the quality of patient position for spinal anesthesia performance (1, unsatisfactory; 2, satisfactory; 3, good; and 4, very good) was evaluated by the anesthesiologist performing it [10]. Moreover, preoperative patient cognitive dysfunction was assessed (0, none; 1, mild; 2, moderate, and 3: severe).

The primary outcome of this study was the percentage of patients who were assessed to have a pain score of intense pain defined as a pain score of 3 or 4 when changing position from the supine to the lateral decubitus position. The secondary outcomes were pain score and heart rate at the four time points, the quality of patient position for spinal anesthesia performance, and the time required for spinal anesthesia (from the start of local anesthesia to the injection of bupivacaine).

### Statistical analysis and sample size calculation

Sample size calculation and all statistical analyses were performed using EZR (ver. 1.41, Saitama Medical Center, Jichi Medical University, Saitama, Japan) [16], which is a graphical user interface for R (The R Foundation for Statistical Computing, Vienna, Austria). Although Lee et al. reported that 76.2% of patients with a hip fracture using fentanyl and dexmedetomidine experienced severe pain when changing positions [14], and the patient population of our hospital is elderly, we estimated the percentage of patients who were assessed to have a pain score of intense pain at the time of lateral positioning to be 50% when the conventional method (fracture side up) was performed, and 60% when the fracture side was down. In setting a non-inferiority margin, i.e., the smallest clinical difference that is acceptable between the two groups, we have relied on our own and outside experts’ clinical judgment to determine that a margin of inferiority of 20% for the difference of the percentage of patients of intense pain between the group is not a significant difference. Because there are no data from previous trials to help define the clinical difference between the positioning pain with the fracture side up and that with the fracture side down. Non-inferiority is demonstrated within the margin of 20% at a one-sided significance level of 0.05 and a power of 80%, with a sample size of 34 per group (68 cases in total). In the present study, assuming a drop-out rate of 15% per side, the final sample size was a set of 78 cases in total.

The data were stored as numerical or categorical data. Continuous variables were summarized as means with standard deviations and medians with interquartile ranges, and categorical data as frequencies with percentages. The patient characteristics and anesthesia/surgical characteristics were assessed using an independent *t* test or the Mann–Whitney *U* test as appropriate, or chi-squared tests. We used chi-squared tests to analyze the primary outcome; an independent *t* test to compare means between the two groups for normally distributed, continuous data (i.e., heart rate, the time required for spinal anesthesia); and a Mann–Whitney *U* test to compare medians for skewed endpoints (i.e., the quality of patient position for spinal anesthesia performance, pain score at the four time points). A value of *p* < 0.05 was considered to indicate a statistically significant difference. All reported *p* values as results are two-sided.

## Results

**Fig. 1:**
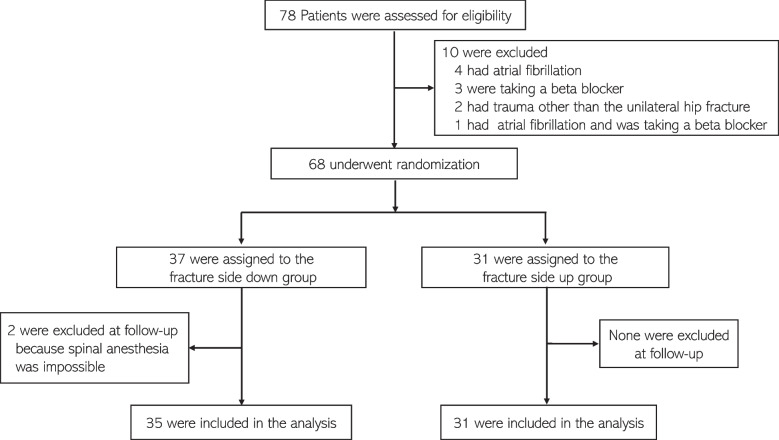
Study Flowchart

A total of 78 patients were met the inclusion criteria for this study (Fig. 1). Ten patients were excluded: 4 patients because of atrial fibrillation; 3 who were taking a beta blocker, 2 who had trauma other than a hip fracture; and 1 who had atrial fibrillation and was taking a beta blocker.

As a result, 68 patients were enrolled and randomly assigned; 37 had been allocated to the fracture side down (left hip fracture) group and 31 to the fracture side up (right hip fracture) group. In the fracture side down group, one patient could not take neither left nor right lateral decubitus position due to pain, and another patient could not perform spinal anesthesia in the left lateral decubitus position due to lumbar deformity. Therefore, the data from 66 patients (35 patients in the fracture side down group and 31 patients in the fracture side up group) were analyzed. The patients’ characteristics are described in Table 1. There were no complications (such as displacement of the fracture area or major hemodynamic changes) caused by position and no conversion to general anesthesia due to inadequate anesthesia in any of the 66 patients.

**Table 1:** Patients’ characteristics

Variables	Fracture side down (*n* = 35)	Fracture side up (*n* = 31)
Age (years)	85 (8)	80 (11)
Weight (kg)	48 (11)	51 (9)
Height (cm)	151 (8)	155 (9)
BMI (kg/m^2^)	21.0 (3.9)	20.9 (2.7)
ASA-PS	2 (2–3)	2 (2–3)
Cognitive dysfunction (0, none; 1, mild; 2, moderate; 3, severe)	2 (1–2)	1 (0–1)

Data are presented as mean (SD) or median (IQR)

*ASA-PS* American Society of Anesthesiologists Physical Status, *BMI* Body mass index, *SD* Standard deviation, *IQR* Interquartile range

### Primary outcome

As shown in Table 2, there were no significant differences between the fracture side down and up groups regarding the percentage of patients who complained of intense pain defined as a pain score of 3 or 4 when changing position from the supine to lateral position; 13/35 (37%) vs 12/31 (39%); 95% confidence interval (95% CI) for the difference of the percentage of patients of intense pain between the groups − 25.0 to 2.2; *p* = 1.000). To be more specific, the pain scores when changing to the lateral position were 0 in 9 patients, 2 in 17, 3 in 25, and 4 in 0.

**Table 2:** Anesthesia/surgical characteristics

Variables	Fracture side down (*n* = 35)	Fracture side up (*n* = 31)	Difference 95% CI	*P* value^a,b^
Intense pain when changing position, *n* (%)	13 (37%)	12 (39%)	− 25.0–2.2	1.00
Quality of patient position (1 unsatisfactory, 2 satisfactory, 3 good, 4 very good)	2 (2–3)	2 (2–3)		0.76
Time required for spinal anesthesia (sec)	172 (150)	185 (158)	− 88.9–63.1	0.74
*Pain score†*				
Baseline at supine position	0 (0–0)	0 (0–0)		0.95
Changing to the left lateral position	2 (1–3)	2 (1–3)		0.84
Local anesthesia was started	0 (0–1)	0 (0–1)		0.82
Puncturing spinal injection was successful	0 (0–0)	0 (0–0)		0.10
*Heart rate (bpm)*				
Baseline at supine position	81 (13)	76 (11)	− 0.4–11.2	0.069
Changing to the left lateral position	86 (14)	79 (12)	− 0.2–12.6	0.057
Local anesthesia was started	81 (12)	75 (10)	0.3–11.5	0.039*
Puncturing spinal injection was successful	80 (13)	73 (10)	1.3–12.9	0.017*
Surgical time (min)	84 (19)	89 (22)	− 14.3–5.7	0.39
Bleeding volume (mL)	143 (97)	147 (113)	− 55.1–48.3	0.90

Data are presented as means (SD) or median (IQR) or counts (percentage)

*SD* Standard deviation, *IQR* Interquartile range, *CI* Confidence interval

^†^Pain was evaluated objectively on a scale of from 0 to 4 (0, calm; 1, facial grimacing; 2, moaning; 3, screaming; 4, unable to proceed because of restlessness or agitation)

^a^*p* value compares fracture side down versus fracture side up

^b^*t* test or Mann–Whitney *U* test used to compare means and chi-squared tests used to compare proportions

### Secondary outcomes

There were no significant differences between the two groups regarding quality of patient position, time required for spinal anesthesia, pain score at any of the four time points, surgical time, and bleeding volume (Table 2). Regarding heart rate, there were no significant differences between the two groups at baseline in the supine position and when changing the position from supine to the left lateral decubitus position. However, the heart rate was significantly higher in the fracture side down group when local anesthesia was started and puncturing spinal injection was successful.

## Discussion

In the present study, there was no significant difference in the percentage of patients with neck fracture assessed to have an intense pain when spinal anesthesia was performed, regardless of whether the fracture side was facing up or down. However, the 95% CI for the difference of patients with intense pain between the groups exceeded the preliminarily set margin of inferiority of 20%; thus, the present study did not demonstrate the non-inferiority of having the fracture side down when performing spinal anesthesia.

Regarding pain whether the fracture side was up or down, we considered that each has advantages and disadvantages. It is generally thought that the lateral decubitus position with the fracture side up was less painful because there was less weight on the fracture site. On the other hand, when a patient has the fracture side down, the movement distance of the fractured part is shortened. The patient is moved from the supine position to the lateral decubitus position by using the lower (fractured) limb as an axis to rotate the body. In addition, the lateral decubitus position with the fracture side down may be more stable than having the fracture side up; thus, the pain caused by instability may be relieved. Therefore, we suspect that the position of the patient does not have a significant effect on the amount of positional pain.

In the lateral decubitus position with the fracture side up, we use isobaric bupivacaine because we are concerned that the effect on the fracture side might be inadequate. However, although non-inferiority of the fracture side down group was not shown, the difference between the two groups was small. Thus, performing spinal anesthesia with the fracture side down may be acceptable. Because of this, placing the affected side down and using hyperbaric local anesthetic are also options. Spinal anesthesia with hyperbaric local anesthetic in the lateral decubitus position naturally works preferentially on the fracture side due to gravity [17]. In addition, performing spinal anesthesia with hyperbaric bupivacaine in the lateral decubitus position with the fracture side down and keeping the patient in the lateral position for a while may reduce hemodynamic changes [18]. Moreover, the use of hyperbaric bupivacaine tends to restore postoperative ambulatory function more quickly than isobaric bupivacaine [19]. Thus, the advantages of using hyperbaric bupivacaine with the fracture side down cannot be ignored. Furthermore, considering the inclination of the lumbar vertebrae, the left lateral decubitus position is usually more comfortable for a right-handed clinician when performing spinal anesthesia [20]. Spinal anesthesia using the paramedian approach is useful for elderly patients with severe spinal deformity. In this situation especially, the needle must be inserted cranially, and it is easy for right-handed clinicians to administer the anesthesia in the left lateral recumbent position. It is clinically advantageous to perform spinal anesthesia in the clinician’s preferred orientation.

In the present study, there was a significant difference between the two groups regarding heart rate at two of the four time points. We included heart rate as a secondary outcome because we thought that an increase in heart rate reflected intense pain. However, the difference between the two groups and the changes during anesthesia in the fracture side down group were small; thus, the differences are not clinically meaningful.

Regarding the percentage of patients who were assessed to have intense pain, it was lower (37–39%) than preliminarily estimated (50–60%) in this study. This result may be because we tried to change the patient’s position with the utmost consideration for pain, which meant the involvement of 4–5 staff members for each change. Calculating the sample size using the rate of complaints of intense pain in this study, non-inferiority would be demonstrated within the margin of 20% at a one-sided significance level of 0.05 and a power of 80%, with a sample size of 90 per group (180 cases in total). However, incorporating 180 patients with a femoral neck fracture into a study is not easy, and collecting data from 180 cases is likely to yield similar results.

There are some limitations to the present study. First, this study was carried out in a single center. Second, the primary outcome was the categorical pain score evaluated by medical staff, which was not a truly objective measurement. The reason for this is that the majority of the patients with a femoral neck fracture are very elderly in Japan; therefore, we considered it not appropriate to set subjective pain evaluation criteria assessed by the patients themselves (e.g., Numerical Rating Scale, Visual Analogue Scale) as the primary outcome. Further studies are required, such as a study in a population that can subjectively and correctly assess pain by themselves, and a study that incorporates an index that may allow objective assessment of pain through the function of the autonomic nervous system (e.g., Analgesia Nociception Index [21], Surgical Pleth Index [22]) as an outcome. Third, the methods of randomization used in the present study were not classic randomization. Fourth, due to the study design, blinding between the two groups was not possible. Fifth, this study did not evaluate the effect of spinal anesthesia. However, in all cases, the surgery was successfully completed without converting to general anesthesia, and this effect is negligible. Sixth, although the present study was a randomized trial, differences between the two groups with regard to cognitive dysfunction may have influenced the results. Finally, considering the risk of displacement of the fracture area due to body position, we did not examine any proximal femoral fractures other than the femoral neck fractures in the present study. However, to the best of our knowledge, there have been no similar studies; therefore, our results are valuable in the sense of reexamining conventionally performed methods, and we hope that our findings will stimulate further research.

## Conclusions

There was no significant difference in the percentage of patients complaining of severe pain when spinal anesthesia was performed on patients with neck fractures, regardless of whether the fracture side was up or down. However, the present study did not reveal non-inferiority of having the fracture side down during performing of spinal anesthesia in patients with a femoral neck fracture.

## Data Availability

The datasets used and/or analyzed during the current study are available from the corresponding author on reasonable request.
